# Trends in Reptile Holdings Across UK Zoos: Identification of the Factors Responsible for Declining Numbers of Venomous Snake

**DOI:** 10.1002/zoo.21868

**Published:** 2024-09-17

**Authors:** Lily Sparrow, Iri Gill, Christopher J. Michaels, Christopher J. Turner

**Affiliations:** ^1^ Department of Life Sciences University of Suffolk Ipswich Suffolk UK; ^2^ Chester Zoo Upton by Chester UK; ^3^ Zoological Society of London, London Zoo London UK

**Keywords:** collection planning, reptile conservation, venomous snakes, zoos

## Abstract

Zoos are under increasing pressure to strategically manage their collections to maximize visitor attendance, financial income, and their contribution to conservation. As a result, the compositions of zoo collections are undergoing significant changes. Many zoos are keeping fewer species and prioritizing keeping large flagship animals that are more attractive to the public. To understand the effects these changes are having on captive reptile numbers, we have analyzed the trends in reptile holdings between 2003 and 2023 at UK zoos. Our findings show that despite an overall increase in reptile numbers in the period analyzed, there has been a dramatic decline in the number of venomous snakes held at UK zoos, and as a result, venomous snakes are being excluded from many of the conservation benefits that zoos provide. To understand the key factors contributing to the decline in venomous snake numbers, 57 staff members across 35 different BIAZA–accredited zoos were surveyed. Results from the survey identified that a perceived increased risk of harm, increasingly stringent health and safety regulations, and increased husbandry requirements were all key contributing factors to why venomous snake numbers at zoos are in decline.

## Research Highlights

- The total number of reptiles kept at UK zoos has increased by 47% between 2003 and 2023.
- UK zoos have increased the number of different reptile genera in their collections.
- Native reptiles comprise less than 2% of the total reptile population held at UK zoos.
- Venomous snake numbers at UK zoos have decreased by 73%, whilst numbers of many other reptile groups, including non‐venomous snakes, have increased.

## Graphical abstract

In this study, we describe the trends in reptile holdings across UK zoos. Our results show that despite an overall increase in reptile numbers, there has been a dramatic decline in the number of venomous snakes being kept at UK zoos, and, as a consequence, venomous snakes are being excluded from many of the conservation benefits that zoos provide.

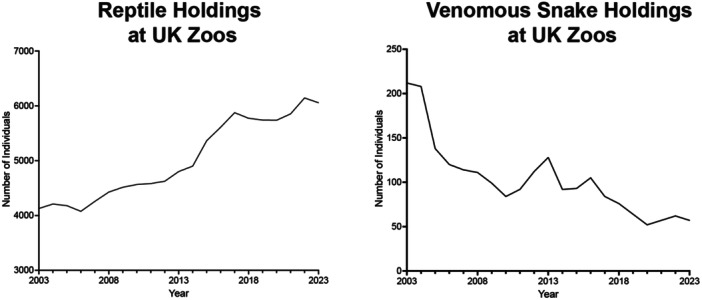

## 1 Introduction

An estimated 21% of all reptiles are thought to be threatened with extinction (Böhm et al. 2013; Cox et al. 2022) due to a combination of climate change, emerging infectious diseases, habitat loss, alien invasive species, persecution, and unsustainable trade (Gibbons et al. 2000; Marshall, Strine, and Hughes 2020; Todd, Willson, and Gibbons 2010). Testudines (turtles, terrapins, and tortoises) and Crocodylia (crocodiles, caimans, alligators, and gharials) have the greatest proportion of threatened species (57.9% and 50%, respectively), followed by Lacertilia (lizards) (19.1%), Serpentes (snakes) (12.2%), and Rhynchocephalia (tuatara) (0%) (Cox et al. 2022). However, despite having a greater number of threatened species than birds and mammals (Cox et al. 2022), reptiles are chronically under‐resourced in terms of conservation and research efforts at a global level (Escribano et al. 2021; Melfi 2009; Rose et al. 2019; Rozzi 2019).

Zoos can play an important role in the conservation of reptiles (IUCN 2023; Miranda et al. 2023). Indeed, several reptile species have been rescued from the brink of extinction due to the expertise and conservation efforts of zoos (Ettling and Schmidt 2015; Smith et al. 2023; Ziegler 2015). One of the primary means that zoos can contribute to the conservation of reptiles is through their collective capacity to breed and maintain viable populations of endangered species that can subsequently be used to restore or bolster wild populations (Andrew et al. 2018; Barbanti et al. 2019; Daltry et al. 2017; Gilbert et al. 2017; Grant and Hudson 2015; Woodfine et al. 2017; Ziegler 2015). Zoos can also use their taxon‐specific expertise and facilities to rescue and rehabilitate species, and raise vulnerable early‐stage reptiles before release, to improve the survivorship of animals in the wild (King and Stanford 2006; Montague 2022; Wijewardena et al. 2023). The presence of reptiles in zoo collections can also help to facilitate research on species that are often too difficult to study in the wild (Rose et al. 2019), and serve as a platform for engaging and educating the public about reptiles to modify behaviors and foster support for broader conservation initiatives and advocate for policy changes (Grajal et al. 2017). This is particularly important for snake conservation as negative attitudes and persecution have directly contributed to their global decline (Gibbons et al. 2000; Vaughn et al. 2022). In addition, zoos can also contribute to reptile conservation by providing financial (Gusset and Dick 2011) and technical support for in situ projects, making them integral to the One Plan approach to the conservation of reptile species (Byers et al. 2013).

Reptiles however are underrepresented globally across zoos and, as a consequence, are losing out on many of the conservation benefits that zoos provide (Brereton and Brereton 2020; Conde et al. 2013; Mooney et al. 2020). Little research has examined the causes of the underrepresentation of reptiles in zoos; however, studies have shown visitors show a strong bias toward mammals and away from culturally maligned groups such as reptiles, for which they typically exhibit neutral to negative attitudes (Colléony et al. 2017; Hutchins, Willis, and Wiese 1995; Mooney et al. 2020; Moss and Esson 2010; Whitworth 2012). Previous studies have shown that visitors have a preference for active, easy‐to‐see species, characteristics not usually associated with reptiles, and dislike animals that lack hair or legs, or are scaly or venomous (Whitworth 2012). Venomous reptiles particularly elicit strong emotions, including fear, disgust, curiosity, and fascination due to their potential danger, which can both attract and repel visitors (Kontsiotis, Rapti, and Liordos 2022; Landová et al. 2020; Marcellini and Jenssen 1988; Rádlová et al. 2019). Since visitor attendance is essential for generating the financial resources required for zoos to fund in situ conservation (Gusset and Dick 2011; Mooney et al. 2020), some authors have urged zoos to take a flagship approach and prioritize keeping large popular mammals to increase visitor attendance and in situ conservation fundraising (Colléony et al. 2017; Hutchins, Willis, and Wiese 1995; Mooney et al. 2020). Large mammals however require large enclosures (reduce the overall carrying capacity of zoos), have less chance of being successfully reintroduced, and are often not well suited to captivity, raising ethical issues that may ultimately deter visitors (Balmford, Mace, and Leader‐Williams 1996; Clubb & Mason 2003). Allocating more space for keeping greater numbers of small‐bodied species with fewer ethical concerns surrounding their captivity may therefore be a more effective strategy for zoos (Balmford, Mace, and Leader‐Williams 1996; Mooney et al. 2020). Others have suggested that zoos should prioritize keeping species with the highest risk of extinction and focus on conserving native species (Conde et al. 2013; Conway 2011; Keulartz 2015; Martin et al. 2014; McCann and Powell 2019). Another potential cause for the underrepresentation of reptiles may be due to the perception that their husbandry and management are more complex compared to other taxa, due to their temperature, humidity, and UV requirements (Goulart et al. 2009). There may also be additional concerns around the safety of keeping venomous reptiles, especially venomous snakes, as their management usually requires close contact as opposed to the remote shunt systems typically used for large predatory mammals (Gill 2006; Mendyk 2023; Smith 2005).

Medically significant venoms are produced by the Helodermatid lizards and members of the Elapidae, Viperidae, Colubridae, and Atractaspididae snake families (Fry et al. 2006). There is also evidence that members of the Varanidae family are venomous; however, the medical and ecological significance of this remains controversial (Dobson et al. 2019; Fry et al. 2006, 2009; Hargreaves, Tucker, and Mulley 2015; Koludarov et al. 2017; Sweet 2016; Weinstein, Smith, and Kardong 2009). Helodermatid lizards include Gila monsters (*Heloderma suspectum*) and the beaded lizards (*Heloderma horridum*). Both are slow‐moving, docile lizards that rarely bite humans in the wild (Beck 2005; Chippaux and Amri 2021). Envenomation by *Heloderma* requires prolonged bites and chewing, which draws venom from glands in the lower jaw onto their long sharp teeth and into the wound (Beck 2005; Mackessy 2022). Bites from *Heloderma* have been documented to cause extreme pain, edema, erythema, hypotension, tachycardia, nausea, and vomiting, but are rarely fatal, despite no antivenom currently being available (Chippaux and Amri 2021). The Elapidae family contains some of the most dangerous species of venomous snakes, including mambas (*Dendroaspis* spp.), taipans (*Oxyuranus* spp.), cobras (*Naja* spp.), kraits (*Bungarus* spp.), and coral snakes (*Micrurus* spp.). Sea snakes (*Hydrophis* spp.) are also commonly included in the Elapidae family, although some consider them to constitute a distinct family. Elapids are fast, agile, and able to inject highly toxic venom with low median lethal dose (LD_50_) values through short fixed front (proteroglyphous) fangs (de la Rosa et al. 2019). The venom of elapid snakes, although a complex of toxins, is primarily neurotoxic (Gutiérrez et al. 2017) and can lead to paralysis and death from respiratory failure if available antivenom is not administered early (Warrell 2010). Several elapid species can also spray cytotoxic venom several meters with amazing accuracy into an individual's eyes, causing intense pain, ophthalmia, and permanent blindness (Gutiérrez et al. 2017; Westhoff, Tzschätzsch, and Bleckmann 2005). Viperidae includes the pit vipers (Crotalinae), true vipers (Viperinae), and Fea's vipers (Azemiopinae). Viperids can inject large volumes of venom in a single strike via long foldable (solenoglyphous) fangs (Hayes et al. 2002). The venoms of viperids are typically hemotoxic and cytotoxic, causing swelling, bleeding, necrosis, and intense pain, and in some species can be neurotoxic (Warrell 2010). Species‐specific (monovalent) and multiple species (polyvalent) antivenoms however, are available for the majority viperid species that can cause fatalities in humans (WHO 2021). The Atractaspididae family contains the venomous *Atractaspis* genus of burrowing asps, also known as mole vipers or stiletto snakes. *Atractaspis* are unique in possessing large protruding hollow movable fangs that allow them to envenom their prey without opening their mouth (Weinstein and Warrell 2019). The fangs have sharp cutting edges and protrude from the closed mouth ventrolaterally allowing them to stab sideways and backwards, making them very difficult to handle safely without the use of anesthesia (Wilkinson 2014). Envenomation by *Atractaspis* is characteristically caused by penetration from a single fang and can cause necrotic, hemorrhagic, and cardiotoxic effects (Weinstein and Warrell 2019). Although rare, several fatalities have been reported following *Atractaspis* envenomation (Tilbury and Verster 2016) and no specific antivenom currently exists (WHO 2021). Although the majority of snakes within the Colubridae family are non‐venomous, or contain venoms that are considered harmless to humans (e.g. *Heterodon* spp.), several species including the boomslang (*Dispholidus typus*), twig snakes (aka vine or bird snakes; *Thelotornis kirtlandi* and *Thelotornis helotornis capensis*), tiger keelback (*Rhabdophis tigrinus*), and red‐backed keelback (*Rhabdophis subminiatus*) are considered dangerous to humans (Weinstein et al. 2013). Venomous colubrids possess rear (opisthoglyphous) fangs, which are not as developed as elapid or viperid fangs and often have to chew or bite their victims for prolonged times to administer medically significant volumes of venom (Weinstein, Warrell, and Keyler 2022). The venoms of colubrids are primarily hemotoxic and can cause coagulopathies, internal bleeding, renal failure, and death (Gutiérrez et al. 2017). Antivenoms are available however, for boomslang and *Rhabdophis*, but not for *Thelotornis* (Weinstein et al. 2013).

Zoos with venomous reptiles also often need to adhere to additional safety requirements determined by their regional zoo legislation. In the United Kingdom, legislation for keeping reptile species classified as posing the greatest risk/hazards (the so‐called “Category 1” species) is enshrined within the Zoo Licensing Act 1981 and includes stringent requirements for enclosure design, staff training, first aid, antivenom availability, and public safety (DEFRA 2012; Gill 2006). Although these are important to minimize the risks associated with working with venomous reptiles, they may also create potential barriers and deter some zoos from keeping venomous reptiles.

To gain further insight into the current underrepresentation of reptiles at zoos, we have analyzed the trends in reptile holdings at UK zoos using the Zoological Information Management System (ZIMS) database and surveyed zoo staff to better understand the causality of the trends.

## 2 Methods

### 2.1 Collection of Trend Data

Reptile holdings at UK zoos from 2003 to 2023 were collated from the Species360 (ZIMS) database between February and March 2023. Medically significant venomous snake genera were identified from the World Health Organization's Snakebite Data Information Portal (WHO 2021) and Appendix 12 of the Secretary of State's Standards of Modern Zoo Practice (DEFRA 2012). Hazardous “Category 1” reptile species posing the greatest risk were identified from DEFRA (2012). Threatened species (categorized as those Critically Endangered, Endangered, and Vulnerable) were identified from the IUCN Red List (IUCN 2022) and Cox et al. (2022).

### 2.2 Questionnaire

Online questionnaires (Data S1) asking participants to share their views on why venomous snake holdings are declining in the United Kingdom were sent to 107 UK BIAZA–accredited institutions and the BIAZA Reptile and Amphibian Working Group. A 1‐month period was given for the questionnaire to be completed with multiple members of staff from each institution permitted to anonymously respond. Responses were received from 57 staff members across 35 different zoos (10 responses were anonymous). The questionnaire consisted of a series of multiple‐choice and Likert scale questions. To avoid question order bias, participants were first asked to provide their opinions on “why venomous snake holdings are declining in the United Kingdom” via an open opinion–based question. Written responses were categorized as relating to: “antivenom”, “conservation,” “cost,” “health and safety,” “risk,” “training,” “visitor interest,” and “other” and could be assigned to more than one category.

The study was approved by BIAZA and the University's research ethics committee.

## 3 Results

### 3.1 Trends in Reptile Numbers at UK Zoos

To understand the current trends in reptile holdings across UK zoos, reptile numbers at UK zoos from 2003 to 2023 were collated from the Species360 (ZIMS) database (Data S2).

**Figure 1:**
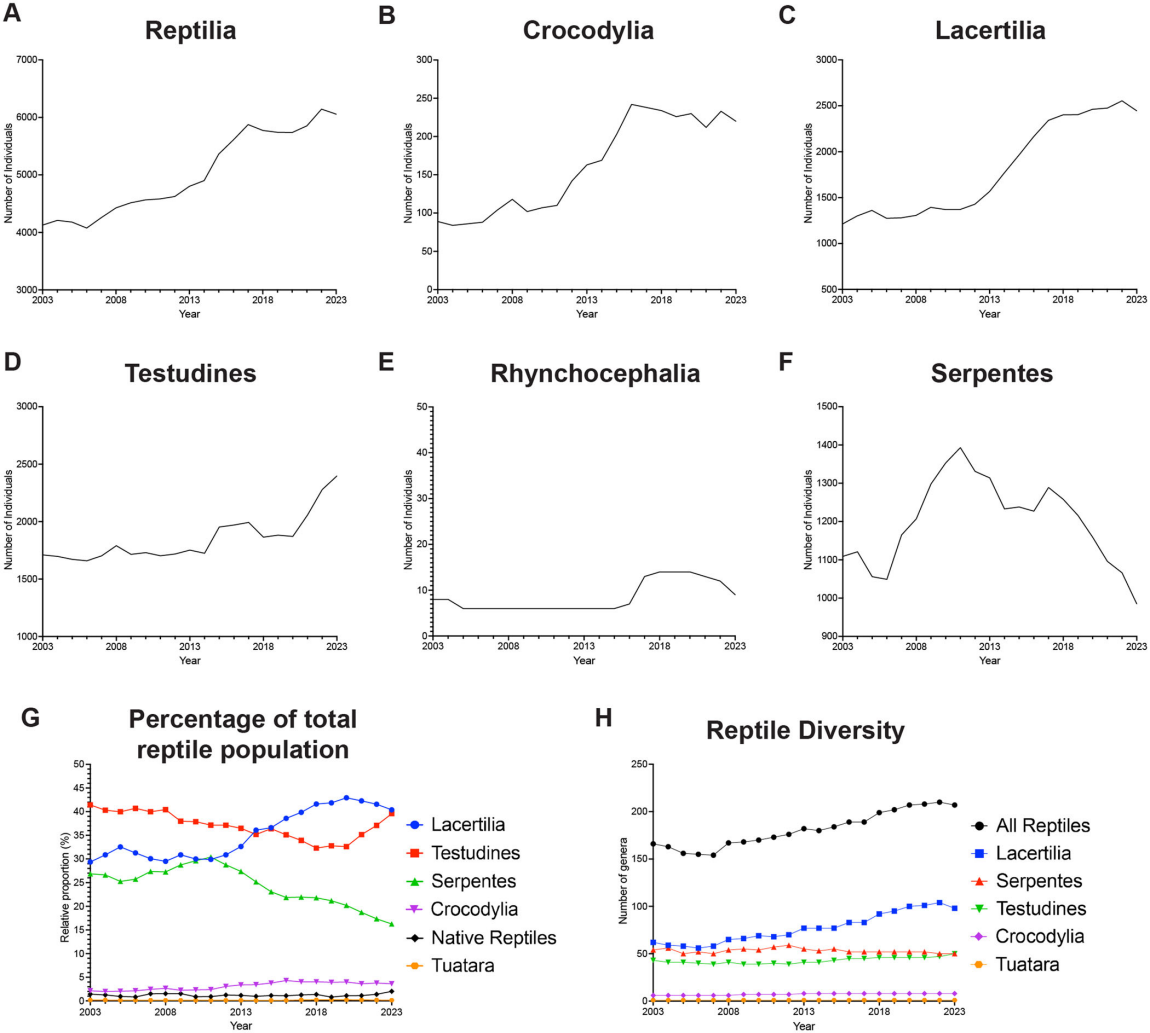
Trends in reptile holdings across UK zoos. (A) Total number of reptiles held across UK zoos between 2003 and 2023. (B–F) Distribution of reptile numbers across the major reptile groups. Numbers of (B) Crocodylia (crocodiles, caimans, alligators, and gharials), (C) Lacertilia (lizards), (D) Testudines (turtles, terrapins, and tortoises), (E) Rhynchocephalia (tuatara), and (F) Serpentes (snakes). No Amphisbaenia were held at UK zoos. (G) Relative holdings of native reptiles and the five major reptile groups in UK zoos between 2023 and 2023. (H) Numbers of different reptile genera represented in UK zoos.

Analysis of the data showed that the total number of reptiles held at UK zoos increased from 4128 to 6057 individuals (47%) between 2003 and 2023 (Figure 1A).

In 2023, 40.4% of reptiles held at UK zoos were Lacertilia, 39.6% were Testudines, with just 16.3% of reptiles being Serpentes, 3.6% Crocodilians, and 0.1% Rhynchocephalia (tuatara) (Figure 1A–G). Amphisbaenians (worm lizards) however were not held at any UK zoos in the period analyzed (Data S2).

Most reptiles held at UK zoos are exotic to the United Kingdom. Numbers of native reptiles fluctuated but made up less than 2% of the total reptile population held at UK zoos between 2003 and 2023 (Figure 1G). In 2023, just four zoos held native species, the majority of which were sand lizards (*Lacerta agilis*) (71 individuals), followed by European adder (*Vipera berus*) (15 individuals), and a single slow worm (*Anguis fragilis*) (Figure S1A and Data S2). No UK zoos held common lizard (*Zootoca vivipara*), grass snake (*Natrix natrix*), or smooth snake (*Coronella austriaca*) at the time of data collection (Data S2).

Data from the ZIMS database also indicated that UK zoos have increased the number of different reptile genera held at their institutions between 2003 and 2023 (Figure 1H). This increase in diversity was largely due to a rise in the number of different Lacertilia genera (62 to 98), as well as increases in the number of Testudines (43 to 50) and Crocodylia (6 to8) genera being held at UK zoos during the period analyzed (Figure 1H).

Crocodilians showed the greatest increase in numbers, rising by 147% since 2003 (Figure 1B), largely due to increased holdings of *Crocodylus*, particularly *Crocodylus niloticus* (Nile crocodile) and the Critically Endangered *Crocodylus siamensis* (Siamese crocodile) (Figure S1B,C).

Lacertilia holdings increased by 102% (Figure 1C) due to increased numbers of Scincomorphs (150%) and Iguanians (122%), as well as smaller increases in the number of Gekkotans (77%) and Platynotans (64%) (Figure S1D). Holdings of Dactyloids (anoles lizards), Phrynosomatids (North American spiny lizards), Diplodactylids (stone geckos), and Shinisaurids (crocodile lizard) in particular rose sharply between 2003 and 2023 (Figure S1E).

Testudines holdings increased by 40% (Figure 1D), largely due to increased numbers of Emydids and Kinosternids (Figure S1F), with holdings of *Pseudemys* (cooters), *Graptemys* (map turtles), and *Sternotherus* (musk turtles) increasing by 3600%, 1780%, and 1567%, respectively (Figure S1G). Holdings of tortoises belonging to the genera *Centrochelys* and *Astrochelys* also displayed marked increases (Figure S1G).

Tuatara were held at just a single institute in the UK between 2003 and 2023. During this period, the numbers remained stable, with a brief increase following successful breeding (the first outside their native New Zealand), before returning to just above their 2003 group size in 2023 (Figure 1E).

In contrast to the other reptile groups, there was a marked shift in Serpentes holdings at UK zoos between 2003 and 2023 (Figure 1F). Following a brief fluctuation in numbers between 2003 and 2007, snake holdings increased from 1165 to a peak of 1393 individuals in 2011 (Figure 1F). From 2011, however, there was a marked reduction in snake holdings at UK zoos (Figure 1F). Between 2011 and 2023, snake numbers decreased by 29% to just 985 individuals, 11% below the population held in 2003 (Figure 1F). In 2011, UK zoos held snakes from 57 different genera, but by 2023, this had reduced to 50 (Figure 1H).

### 3.2 Declining Numbers of Venomous Snakes at UK Zoos

**Figure 2:**
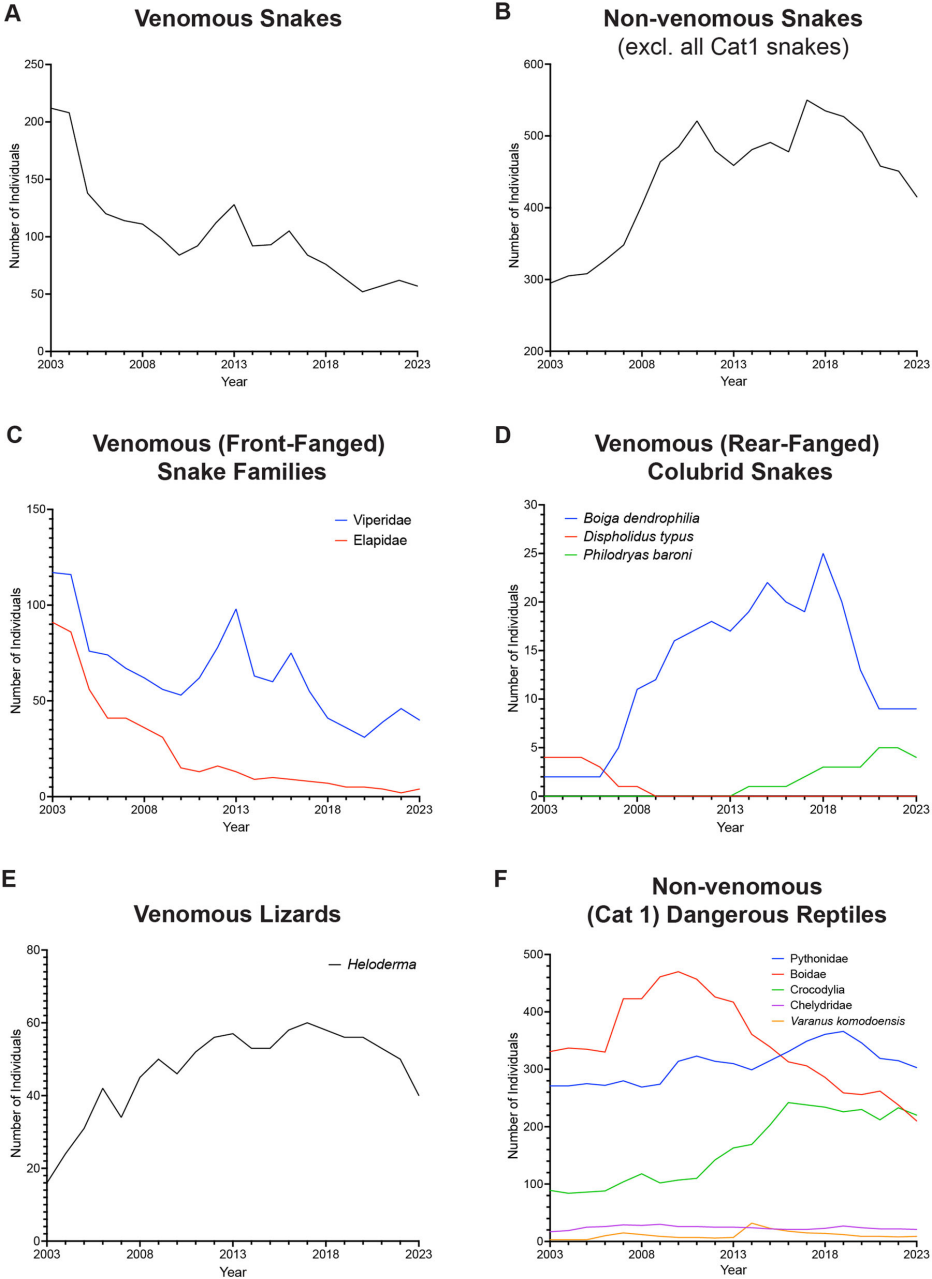
Declining numbers of venomous snakes at UK zoos. Number of (A) venomous and (B) non‐venomous snakes (excluding non‐venomous Category 1 snakes) held across UK zoos between 2003 and 2023. Trends in holdings of (C) front‐fanged venomous and (D) rear‐fanged venomous snakes. Trends in holdings of (E) medically significant venomous lizards and (F) non‐venomous Category 1 classified dangerous reptiles across UK zoos.

Further analysis of the snake holding data revealed that medically significant venomous snakes (hereafter referred to as venomous snakes) had undergone the greatest reduction in numbers between 2003 and 2023. Venomous snake holdings decreased by 73% between 2003 and 2023 (Figure 2A). In 2003, UK zoos held venomous snakes from 20 different genera, but by 2023, only 10 genera were represented (Figure S2A).

In contrast, holdings of non‐venomous (non‐Category 1) snakes increased by 40% (Figure 2B), largely through increases in the number of *Pantherophis* (corn snakes, 137 to 156), *Lampropeltis* (king snakes, 58 to 73), and the non‐medically significant venomous *Heterodon* (hognosed snakes, 5 to 31), and *Thamnophis* (garter snakes, 1 to 35) (Data S2). In 2003, 34 non‐venomous snake genera were represented in UK zoos, but by 2023, 40 genera were held (Figure S2B).

Elapids showed the greatest decline. In 2003, 91 elapid snakes were held in UK zoos, but by 2023, this number had reduced to just four individuals (a reduction of 96%) (Figure 2C) with just *Naja pallida* (red spitting cobra) and a single *Ophiophagus hannah* (king cobra) remaining in captivity (Figure S2C).

Viperid holdings also showed a marked decline (Figure 2C), decreasing from 117 to 40 individuals (66%) between 2003 and 2023 (Figure 2C). In 2003, viperids from 13 different genera were held at UK zoos; however, by 2023, just six genera remained (Figure S2D), and only two institutions held European adder, the United Kingdom's only native venomous snake species (Data S2).

The total number of venomous rear‐fanged colubrid snakes held at UK zoos increasedhowever, largely due to a rise in the number of *Boiga dendrophilia* (mangrove snake), which increased from 2 to 25 between 2003 and 2018 before declining sharply and stabilizing at nine individuals from 2021 to 2023, and to a lesser extent through the introduction and rise in numbers of *Philodryas baroni* (Baron's green racer) in UK zoos (Figure 2D). It is worth noting, however, that *Dispholidus typus* (boomslang), which possesses highly toxic venom, was phased out of captivity in the United Kingdom during the period analyzed, dropping from four individuals in 2003 to no longer being held by 2009 (Figure 2D). Furthermore, no zoos held twig snakes (*Thelotornis kirtlandi*, *Thelotornis capensis*), the tiger keelback (*Rhabdophis tigrinus*), or the red‐backed keelback (*Rhabdophis subminiatus*), which are also viewed as dangerous rear‐fanged species, nor any venomous atractaspidid species (Data S2).

Interestingly, holdings of *Heloderma* lizards, which also possess medically significant venom, also increased in the period analyzed (Figure 2E). Furthermore, with the exception of boas, the number of non‐venomous hazardous/high‐risk Category 1 reptiles (as classified by DEFRA, 2012), including pythons, Chelydridae (snapping turtles), Crocodylia, and Komodo dragons (*Varanus komodoenesis*), increased in the same period (Figure 2F).

### 3.3 Identification of the Factors Responsible for Declining Numbers of Venomous Snake

To understand the key factors contributing to the decline in venomous snake numbers at UK zoos, 57 staff members across 35 different zoos (10 responses were anonymous) were surveyed (Data S1).

Sixty‐five percent of respondents did not hold venomous snakes at their organization (hereafter referred to as Non‐Holders), with 12% of respondents having not held venomous snakes in the last 50 years (Figure S3A). Interestingly, 35% of Non‐Holders and all venomous snake holders (hereafter referred to as VS Holders) surveyed held non‐venomous snakes at their organization (Figure S3B).

### 3.4 Zoos With Venomous Snakes in Their Collection Contribute More to Venomous Snake Conservation, Education, and Research

**Figure 3:**
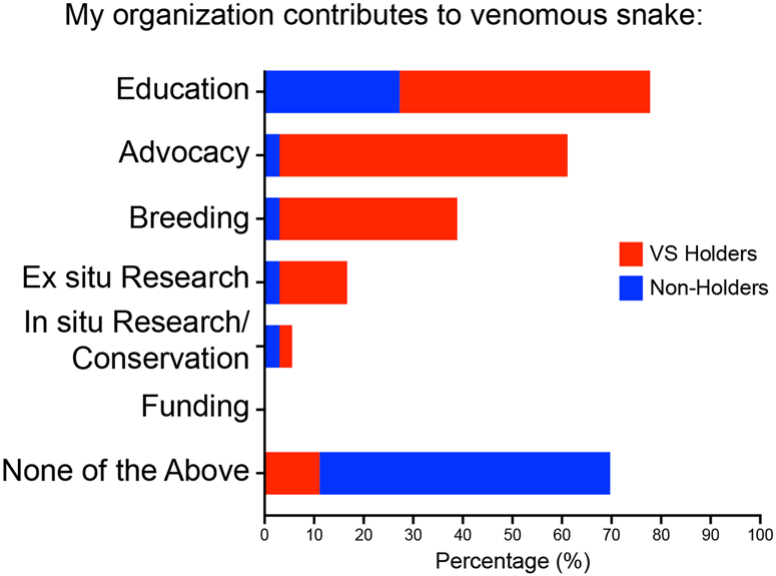
Contribution to venomous snake conservation, education, and research by UK zoos. Note that zoos with venomous snakes within their collection (VS Holders) are more likely to contribute to venomous snake conservation, education, advocacy, and research than zoos without venomous snakes (Non‐Holders).

Eighty‐nine percent of VS Holders surveyed contribute to some form of venomous snake conservation, education, or research (Figure 3). In contrast, just 30% of Non‐Holders surveyed make any contribution (Figure 3). Keeping venomous snakes at UK zoos appears particularly important for supporting venomous snake advocacy, education, and ex situ research, increasing each by 58%, 51%, and 14% respectively (Figure 3). Interestingly, only 6% of VS Holders surveyed contribute to in situ venomous snake conservation or research (just 3% more than Non‐Holders), and none of the zoos surveyed provided funding specifically for venomous snake conservation (Figure 3).

### 3.5 Key Factors Contributing to the Decline in Venomous Snake Holdings in UK Zoos

**Figure 4:**
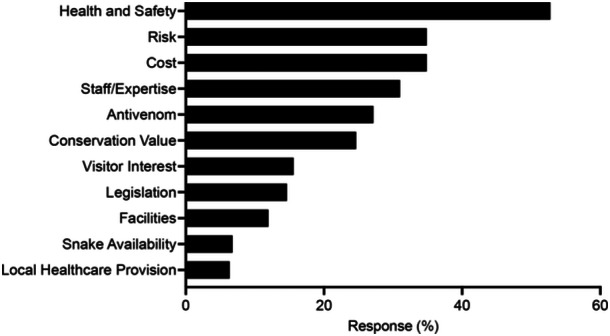
Factors contributing to the decline in venomous snake holdings in UK zoos. Responses to the open text question “Why are venomous snake holdings declining in the United Kingdom?” identified 11 contributing factors.

To identify factors contributing to the decline in venomous snake holdings at UK zoos, participants were first asked to describe why they think holdings are declining. Categorization of the text responses identified 11 potential contributing factors (Figure 4). The most suggested factors from both VS Holders and Non‐Holders were related to the need to comply with health and safety regulations (53%), increased risk of harm (35%), increased costs (35%), and not having sufficient numbers of staff trained in venomous snake husbandry (31%) (Figure 4). Additional factors identified were categorized as relating to complications around holding antivenom (27%), conservation importance/value (25%), lack of visitor interest (16%), legislation (15%), facilities (12%), availability of venomous snakes (7%), and adequate local healthcare provision (6%) (Figure 4).

To gain further insight and assess the extent to which each factor contributed to the decline, participants were asked a series of closed‐ended questions relating to the identified categories.

**Figure 5:**
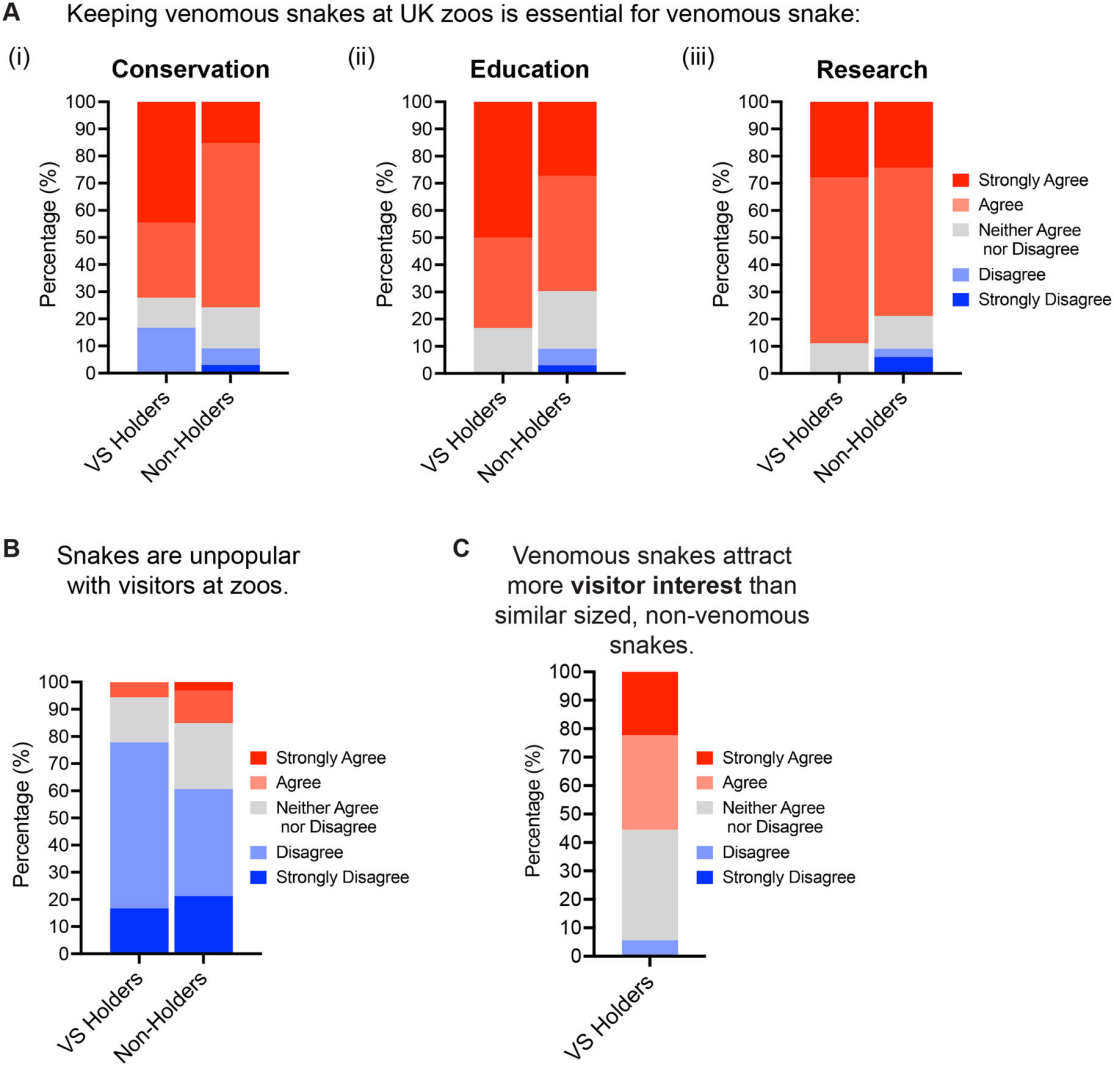
Perceived conservation value and visitor interest in venomous snakes held at UK zoos. (A) Percentage of respondents who agree (or disagree) that holding venomous snakes at UK zoos is essential for venomous snake (i) conservation, (ii) education, and (iii) research. (B) Percentage of respondents who believe that snakes are unpopular with visitors at zoos. (C) Percentage of respondents who believe that venomous snakes attract more visitors than similarly sized non‐venomous snakes.

Analysis of the results showed that neither a perceived lack of conservation value nor a perceived lack of visitor interest are major contributing factors to the decline in venomous snake holdings in the United Kingdom (Figure 5). Seventy‐two percent of VS Holders and 76% of Non‐Holders agreed that keeping venomous snake at UK zoos is essential for venomous snake conservation (Figure 5A). Furthermore, the majority of VS Holders and Non‐Holders agreed that holding venomous snake at UK zoos was also essential for venomous snake education and research (Figure 5A). Despite previous studies indicating that snakes are less charismatic and attractive to visitors, just 6% of VS Holders and 15% of Non‐Holders believed that snakes are unpopular with visitors at zoos (Figure 5B). Furthermore, 55% of VS Holders (all of whom also held non‐venomous snakes) agreed that venomous snakes attract more visitor interest than similarly sized non‐venomous snakes at their zoo, with just 6% disagreeing (Figure 5C).

**Figure 6:**
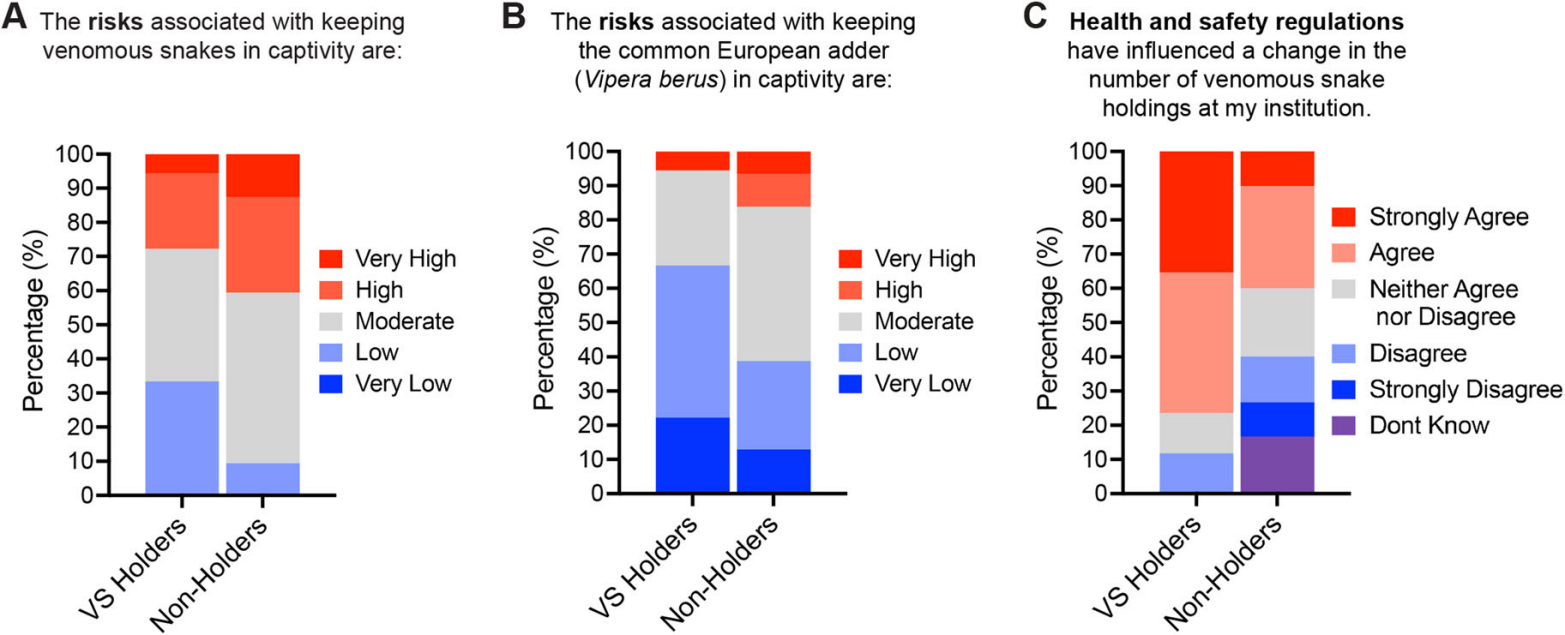
Health and safety concerns are a major cause of the decline in venomous snake numbers at UK zoos. Perceived risk of keeping venomous snakes (A), and specifically European adder (*Vipera berus*) (B) in captivity. (C) Influence of health and safety regulations on venomous snake holdings at UK zoos.

Survey results however confirmed that the perception that captive venomous snake pose a high risk combined with the need to comply with health and safety regulations are key factors contributing to the decline (Figure 6). Non‐Holders were more likely to rate the risk of keeping venomous snakes as being moderate, high, or very high, with just 9% of Non‐Holders rating the risk as low, compared to 33% of VS Holders (Figure 6A). To confirm that this difference was not simply due to Non‐Holders familiarity with only the most notorious/dangerous venomous snakes, participants were asked to assess the risk associated with keeping the common European adder, a native venomous snake species known to both groups. Interestingly, the proportion of participants rating the risk as low or very low increased in both groups; however, Non‐Holders were again more likely to rate the risk as moderate, high, or very high compared to VS Holders (Figure 6B), confirming that Non‐Holders perceived a greater risk in keeping venomous snakes in captivity than those currently holding venomous snakes. Furthermore, 76% of VS Holders and 40% of Non‐Holders agreed (12% and 23% disagreed, respectively) that health and safety regulations have influenced a change in the number of venomous snake holdings at their institution (Figure 6C).

**Figure 7:**
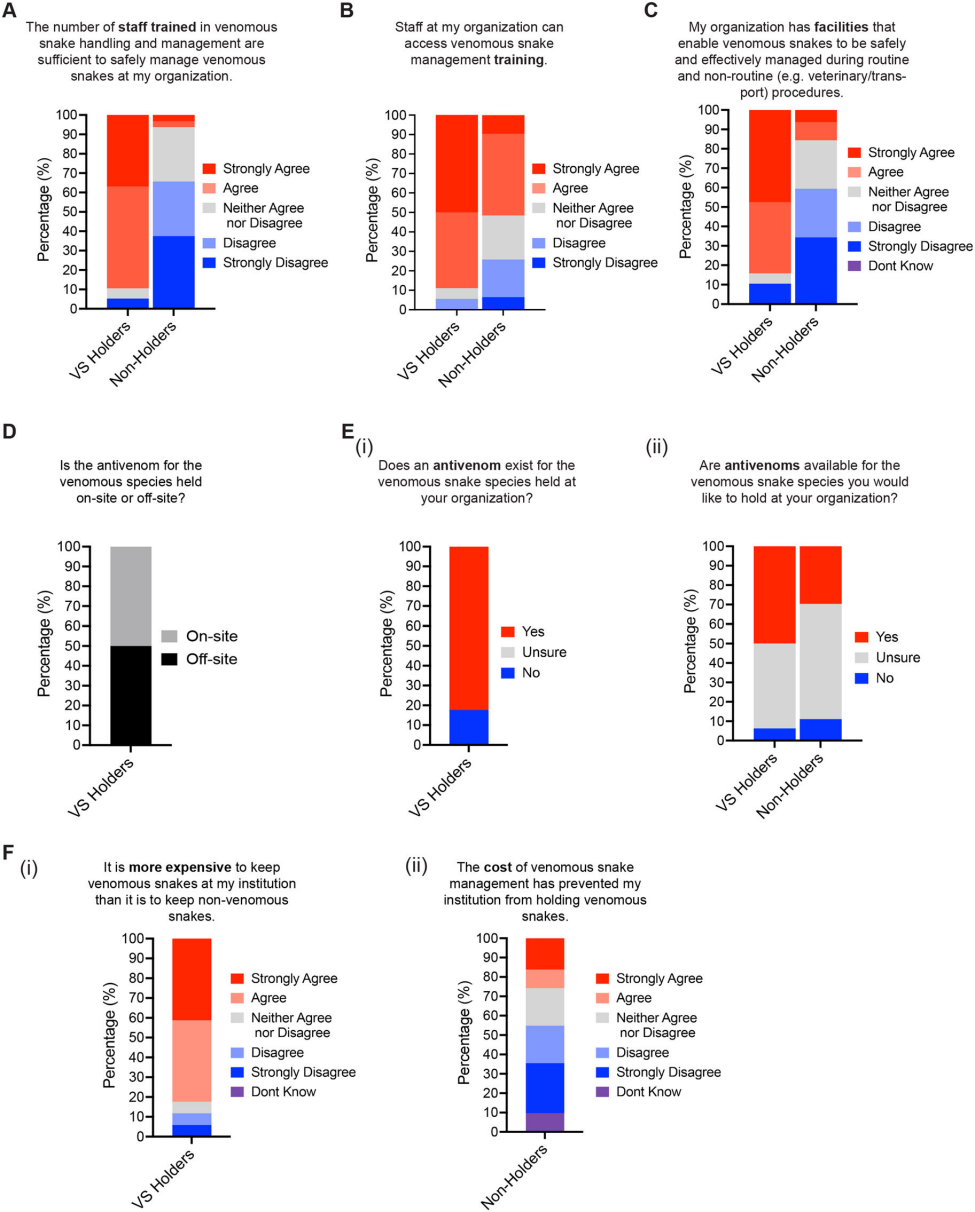
Venomous snake management requirements affecting venomous snake holdings. Survey responses to questions relating to: (A) staff expertise, (B) staff training, (C) facilities, (D) antivenom storage, (E) antivenom availability for (i) species currently held and (ii) species respondent would like to hold, (F) costs of venomous snake management (i) being more expensive compared to non‐venomous snakes and (ii) preventing the respondents from keeping venomous snakes.

Data from the survey also confirmed that the additional husbandry requirements needed to keep venomous snakes safely are also key factors contributing to the decline (Figure 7). Only 6% of Non‐Holders had a sufficient number of staff trained in venomous snake handling and management to keep venomous snakes (Figure 7A), despite 52% agreeing that staff at their organization can access venomous snake training (Figure 7B). Furthermore, 57% of Non‐Holders felt they did not have facilities that enable venomous snakes to be safely and effectively managed during routine and non‐routine procedures at their organizations (Figure 7C). Although 84% of VS Holders agreed that their organization has sufficient facilities to manage venomous snakes, 11% (strongly) disagreed (Figure 7C), and half of all the VS Holders surveyed stored their antivenom at off‐site facilities (Figure 7D).

The availability of antivenom was also confirmed as a contributing factor (Figure 7E). Only 18% of VS Holders kept venomous snake species for which no antivenom is currently available (Figure 7E). A large proportion of respondents (44% VS Holders and 59% of Non‐Holders) were unsure whether antivenom is available for venomous species they would like to hold at their organization in the future; however, 50% of VS Holders and 30% of Non‐Holders stated that antivenoms are currently available (Figure 7E).

Finally, we asked whether the additional husbandry requirements associated with holding venomous snakes made them more expensive to keep. Eighty‐two percent agreed that venomous snakes are more expensive to hold compared to non‐venomous snakes (Figure 7F). Furthermore, 26% of Non‐Holders agreed (16% strongly) that the increased cost had prevented their institution from holding venomous snakes (Figure 7F). Forty‐five percent of Non‐Holders however disagreed (26% strongly) that the cost of venomous snake management had prevented their institution from holding venomous snakes, suggesting that the increased cost may be a key factor for some but not all organizations.

## 4 Discussion

Our study shows that UK zoos have drastically reduced the number of venomous snakes they keep in their collections (Figure 2A), and, as a consequence, venomous snakes are losing out on the many conservation benefits that zoos provide (Figure 3).

In the last 20 years, venomous snake numbers at UK zoos have decreased by 73%, whilst numbers of many other reptile groups, including non‐venomous snakes, have increased (Figures 1 and 2). As a result, there are now more privately owned venomous snakes in the United Kingdom than held in UK zoos (400 vs. 57) (Born Free 2023). In 2003, venomous snakes from 20 different genera were represented in UK zoos; however, by 2023, just 10 remained (Figure S2A). At the time of data collection, just 6% of all snakes held in UK zoos were venomous. Interestingly, similar declines have been observed at AZA–accredited institutions (Peeling 2016), suggesting that this may be indicative of much wider regional declines in venomous snake holdings.

Results from our survey identified several contributing factors for the decline in venomous snake holdings at UK zoos (Figure 4). A major cause for the decline was the requirement of zoos to meet increasingly stringent health and safety regulations and where possible reduce or eliminate risks from their institutions. In response, many zoos have reduced or removed venomous snakes, which are often perceived as posing a greater risk than other taxa (Mendyk 2023), from their collections and replaced them with safer alternatives. Analysis of holding data suggests that many zoos are replacing their venomous snakes with non‐venomous snakes, particularly those with calm temperaments such as corn and king snakes that can be used in live handling displays and also act as a proxy for more dangerous species (Kerr 2021). Previous studies have shown that direct interaction with live animals at zoos can increase visitor knowledge and connectivity to animals and can reduce negative attitudes toward snakes (Stanford 2014; Wünschmann et al. 2017). Keeping safe‐to‐handle snake species in preference to venomous snakes may therefore be a better strategy to educate the public about snake conservation. However, some argue against the effectiveness of such activities and have ethical and welfare concerns about using animals in live handling displays and encounters at zoos (Spooner et al. 2021). The similarity in appearance of corn snakes and king snakes to the venomous copperhead and coral snakes, respectively, can also provide opportunities to educate visitors about venomous snakes and highlight how misidentification can lead to the persecution of harmless species (Valkonen et al. 2018), without the need to keep venomous snakes at their institutions. Indeed, 27% of Non‐Holders surveyed contributed to venomous snake education without having any venomous snakes in their collections (Figure 3). Holding data also indicates that zoos are reducing risks by choosing to keep non‐medically significant venomous snakes, particularly hognose and garter snakes, rather than medically significant species. Additionally, zoos are keeping greater numbers of rear‐fanged snakes in their collections (Figure 2D). Interestingly, despite also possessing medically significant venom, holdings of Helodermatid lizards increased in the period analyzed (Figure 2E). This is most likely due to *Heloderma* being perceived as less of a safety risk due to being easier to handle and less likely to cause fatal bites in healthy adults than venomous snakes (Beck 2005; Chippaux and Amri 2021).

The additional husbandry requirements needed to keep venomous snakes safely are also key factors contributing to the decline (Figure 7). Current legislation and guidelines in the United Kingdom require that zoos with venomous snakes have specialist facilities that enable the safe routine management of venomous snakes and sufficient staff with expertise in venomous snake handling available at all times (DEFRA 2012). Zoos with venomous snakes are also required to have readily accessible up‐to‐date antivenom, on‐site at the zoo or off‐site at a hospital (DEFRA 2012). Since most venomous snakes kept at UK zoos are exotic (Figure 1G), antivenoms for most species are often not available at local medical facilities and must be bought and stored by zoos in strict accordance with the manufacturer's instructions and replaced before expiration regularly. Antivenoms can be extremely costly; for example, a polyvalent antivenom for four North American pit viper species (Crofab) costs >£2500 a vial, with potentially 12 vials (or more) required for a single bite (Mazer‐Amirshahi, Stolbach, and Nelson 2018). Alongside the need for additional refrigeration facilities, and licenses to hold antivenom on site, this may be unaffordable for some small to medium sized zoos with smaller visitor numbers and less external funding (Figure 7F). In addition to cost, some zoos were also concerned that even when effective antivenom was available, local health providers lacked experience in treating non‐native venomous snake bites (Data S1).

The rise in holdings of crocodiles, pythons, and Komodo dragons (Figure 2F) suggests that UK zoos are also choosing to prioritize the keeping of large “flagship” reptile species over smaller venomous snakes to increase visitor attendance and ultimately income for conservation and research (Mooney et al. 2020). Previous studies have shown that large‐bodied animals attract more visitors than small‐bodied species (Moss and Esson 2010; Ward et al. 1998; Whitworth 2012). Indeed, despite being inactive for most of the day, crocodiles have above‐average visitor holding times and attracting power (Marcellini and Jenssen 1988; Moss and Esson 2010), which may partly explain why they have undergone the greatest percentage increase in numbers among all the reptile groups (Figure 1B). Like venomous snakes however, many of these large “flagship” reptiles are classified as high‐risk (Category 1) animals and have significant additional husbandry requirements. Large “flagship” reptiles also require large enclosures and as a result take up a lot of valuable space that could be used to house a greater number of enclosures for smaller reptiles (Balmford, Mace, and Leader‐Williams 1996; Mooney et al. 2020). Although venomous snakes cannot be held in mixed exhibits as easily as tortoises and lizards, most venomous snakes do not require large enclosures and can have a significant conservation impact while taking up little valuable space at zoos (Peeling 2016). Our results also suggest that collection planners may perhaps be underestimating the popularity of venomous snakes. In agreement with previous observations (Marcellini and Jenssen 1988), 55% of VS Holders agreed (6% disagreeing) that venomous snakes attract more visitor interest than similar sized non‐venomous snakes (Figure 5C).

The underrepresentation of venomous snakes in UK collections may also be due to zoos focusing their efforts on managing more threatened taxa. Recent analysis has shown that with the exception of tuatara, snakes have the lowest proportion of threatened species out of all the major reptile groups (Cox et al. 2022). This is reflected in our holding data, which shows increases in the numbers of turtles, crocodiles, and lizards but not snakes, in UK zoos (Figure 1). It could be argued, however, that due to snakes having a greater number of threatened species than turtles and crocodiles (Cox et al. 2022), venomous snakes should be better represented in European breeding programs. Furthermore, 75% of all respondents in our survey agreed that keeping venomous snakes at UK zoos is essential for their conservation (Figure 5A).

The decline in venomous snake holdings at UK zoos will significantly reduce the contribution that UK zoos make to venomous snake conservation. Results from our study show that in addition to captive breeding, zoos without venomous snakes are less likely to contribute to venomous snake education, advocacy, and research than those with venomous snakes in their collection (Figure 3).

Improving knowledge and public attitudes toward snakes is particularly important for snake conservation as the killing of venomous and harmless misidentified non‐venomous snakes by humans is a major threat to snakes in the wild (Gibbons et al. 2000; Vaughn et al. 2022). Educating the public about the challenges and risks of keeping venomous snakes at home and promoting responsible pet ownership is also becoming increasingly important. A rise in the popularity of keeping exotic pets in the United Kingdom has led to increased numbers of exotic venomous snake bite incidents and concerns about the welfare of snakes kept in private homes due to inadequate husbandry and a lack of veterinarians with venomous snake expertise (Azevedo et al. 2021; Cargill, Benato, and Rooney 2022; Born Free 2023; Jagpal et al. 2022; Loeb and Leeming 2020). Since venomous snakes are still mainly sourced from the wild, there is also concern that the rise in pet trade will impact wild populations, put catchers' lives at risk, and increase the chance of introducing diseases that can threaten native wildlife and public health (Hierink et al. 2020).

The loss of research on what is already an under‐investigated group (Melfi 2009; Rose et al. 2019) will also have a significant impact. Captive populations provide opportunities to carry out research into the behavior, biology, and diseases of venomous snakes that are often difficult to conduct in the wild (Murphy 2014a, 2014b; Rose et al. 2019). Furthermore, zoo collections of venomous snakes can provide valuable opportunities for venom extraction, antivenom production, and drug discovery for human benefit (Oliveira et al. 2022). With fewer institutions maintaining venomous snakes in their collection, the number of zoo staff with venomous snake expertise in the United Kingdom will also decline, reducing the ability of zoos to provide taxon‐specific expertise to support research and conservation projects in the wild. The loss of expertise may also accelerate the decline in holdings and prevent zoos from keeping venomous snakes in the future. Indeed, a lack of sufficient numbers of staff trained in the husbandry and management of venomous snakes was a key factor why some zoos are unwilling to keep venomous snakes, despite training being available (Figures 4 and 7A,B). A decline in the number of institutions willing to manage venomous snakes will also reduce the collective global carrying capacity for the ex situ conservation of venomous snake species. This may result in zoos focusing their efforts on managing fewer species to ensure they can establish sustainable genetically diverse populations and the loss of many important venomous snake species from captivity. Interestingly, a lack of availability of venomous snakes was identified as a key factor for the decline in venomous snake holdings at UK zoos (Figure 4).

## 5 Conclusion

In conclusion, our results show that there has been a dramatic decline in the number of venomous snakes held at UK zoos, and as a result, venomous snakes are being excluded from many of the conservation benefits that zoos provide. Our data suggest that increased health and safety risks and increased husbandry requirements, especially the need for expensive antivenom, were all key contributing factors to why venomous snake numbers at zoos are in decline.

To overcome these barriers and increase venomous snake holdings and conservation, we propose that UK zoos consider adding the European adder (*Vipera berus*) to their collections. European adders are the UK's only venomous snake species, they are held at only a small number of UK zoos, and they are in desperate need of conservation. Recent data have shown that 90% of European adder populations in Britain are in decline and by 2032 could be extinct in the United Kingdom (Gardner, 2019; Milton 2022). By increasing holdings of European adder, UK zoos can educate the public about the ecological importance of venomous snakes, reduce persecution, and foster support for wider conservation initiatives (Kelly et al. 2023). Helping the public to identify European adders may also reduce the persecution of the non‐venomous native grass snake, and the rare smooth snake that looks similar in appearance to adders (Valkonen et al. 2018). Keeping European adders more widely across UK zoos will also secure an insurance population and facilitate conservation breeding for possible reintroduction projects in the future. Furthermore, European adders are small snakes, averaging 40–70 cm in length, so they will take up very little of the valuable space at zoos and are relatively inexpensive to keep. Antivenom for adder bites is inexpensive relative to antivenoms for non‐native species and is more widely available across UK hospitals, which are more experienced in treating adder envenomation compared to bites from exotic species. Furthermore, their venom toxicity and yields are comparatively low compared to many other venomous snakes (Warrell 2005), and an estimated 30% of adder bites are “dry,” where the snake does not inject venom (Valenta et al. 2014), making them far less of a risk than many other venomous species. Indeed, just 12% of respondents surveyed thought that European adders posed a high risk (Figure 6B). Increasing the number of collections that house European adders will also increase expertise in venomous snake husbandry and management across the United Kingdom. This will enable greater numbers of zoos to rescue and rehabilitate adders, contribute to venomous snake research, and manage endangered species in the future.

## Supporting information

Supplemental Data 1 | Survey questions and anonymized responses.

Supplemental Data 2 | Reptile holdings at UK zoos between 2003 and 2023.

Supplemental Figure 1 | Breakdown of reptile holdings in UK zoos. (A–G) Trends in holdings of (A) reptile species native to the United Kingdom, (B) *Crocodillian* genera, (C) *Crocodylus* species, (D) *Lacertilia infraorders*, (E) Lacertilia families (with > 100% increase in holdings), (F) Testudines families, and (G) *Testudines* genera (with > 100% increase in holdings).

Supplemental Figure 2 | Venomous snake holding trends at UK zoos. Number of different (a) venomous and (b) non‐venomous snake genera represented at UK zoos. Trends in holdings of (C) *Elapidae* and (D) *Viperidae* genera at UK zoos between 2003 and 2023.

Supplemental Figure 3 | Demographic of survey respondents. (A) Percentage of survey respondents that hold or have held venomous snakes at their institute. (B) Percentage of VS Holders and Non‐Holders that hold non‐venomous snakes at their organization.

## Data Availability

All raw data used in this study are available in the Supporting Information.
