# Supplementary material for: Trends in Reptile Holdings Across UK Zoos: Identification of the Factors Responsible for Declining Numbers of Venomous Snake
Source: Zoo Biol. 2024 Sep 17;43(6):556–69. doi: 10.1002/zoo.21868 (PMC11624627; doi:10.1002/zoo.21868)
Supplement: Supplementary file 3 — Supplemental Figure 1 | Breakdown of reptile holdings in UK zoos. (A–G) Trends in holdings of (A) reptile species native to the United Kingdom, (B) Crocodillian genera, (C) Crocodylus species, (D) Lacertilia infraorders, (E) Lacertilia families (with > 100% increase in holdings), (F) Testudines families, and (G) Testudines genera (with > 100% increase in holdings). [file ZOO-43-556-s004.pdf]

Supplemental Figure 1.

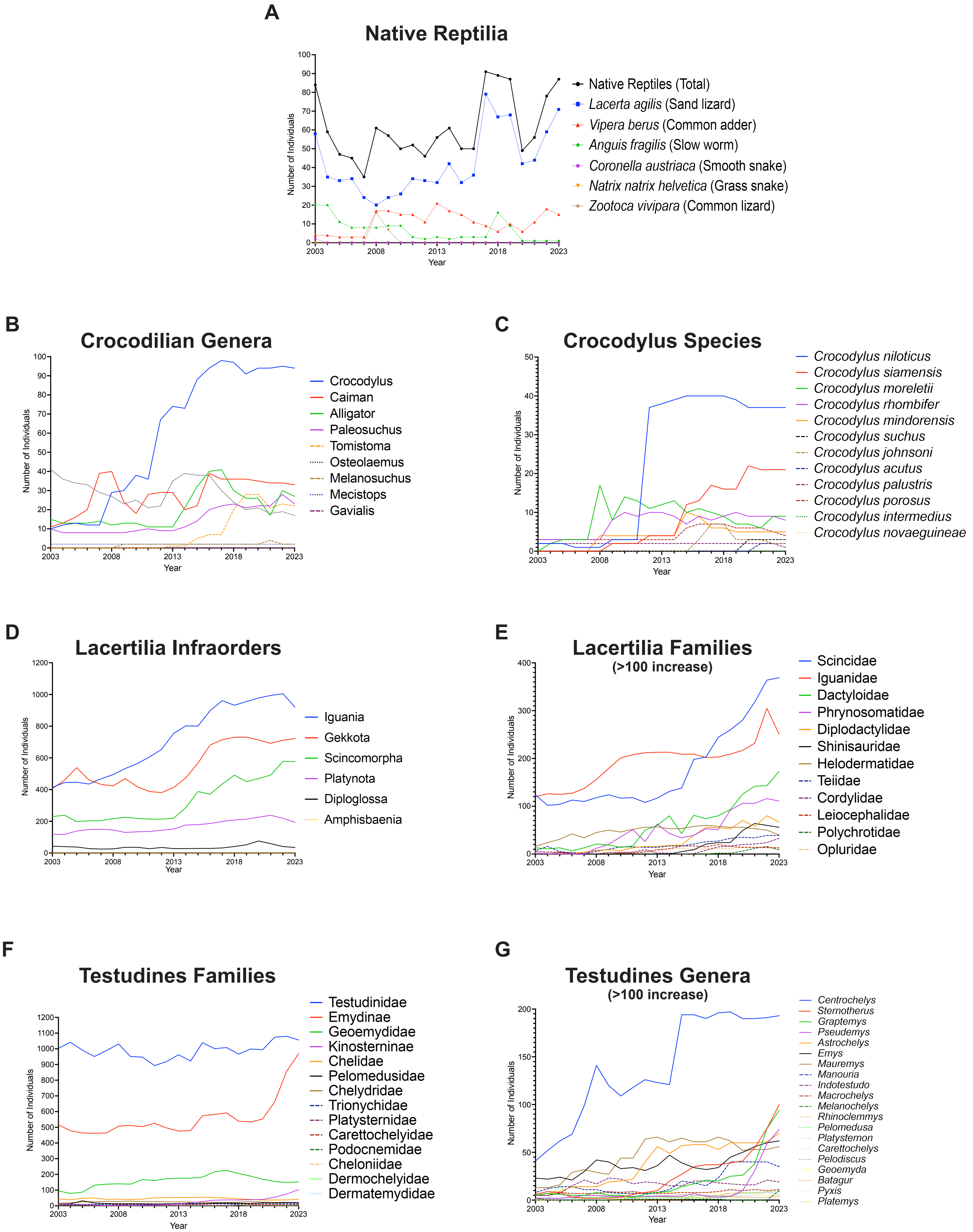

Supplemental Figure 1. Breakdown of Reptile Holdings in UK Zoos. (A-G) Trends in holdings of (A) Reptile species native to the UK, (B) Crocodillian genera, (C) Crocodylus species, (D) Lacertilia infraorders, (E) Lacertilia families (with >100% increase in holdings), (F) Testudines families, and (G) Testudines genera (with >100% increase in holdings).
