# Supplementary material for: Trends in Reptile Holdings Across UK Zoos: Identification of the Factors Responsible for Declining Numbers of Venomous Snake
Source: Zoo Biol. 2024 Sep 17;43(6):556–69. doi: 10.1002/zoo.21868 (PMC11624627; doi:10.1002/zoo.21868)
Supplement: Supplementary file 4 — Supplemental Figure 2 | Venomous snake holding trends at UK zoos. Number of different (a) venomous and (b) non‐venomous snake genera represented at UK zoos. Trends in holdings of (C) Elapidae and (D) Viperidae genera at UK zoos between 2003 and 2023. [file ZOO-43-556-s005.pdf]

Supplemental Figure 2.

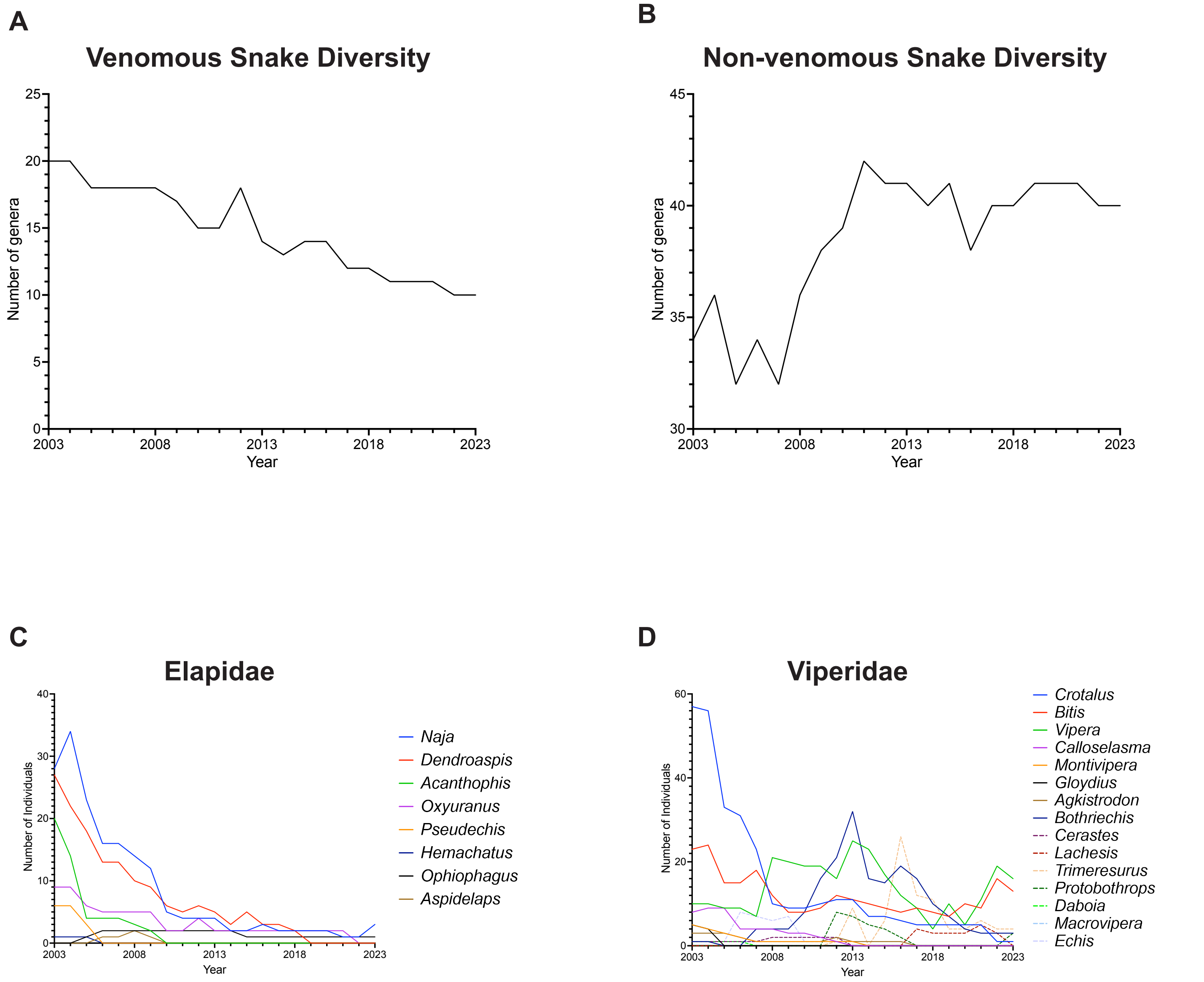

Supplemental Figure 2. Venomous snake holding trends at UK zoos. Number of different (A) venomous and (B) non-venomous snake genera represented at UK zoos. Trends in holdings of (C) Elapidae and (D) Viperidae genera at UK zoos between 2003 and 2023.
