# Supplementary material for: Trends in Reptile Holdings Across UK Zoos: Identification of the Factors Responsible for Declining Numbers of Venomous Snake
Source: Zoo Biol. 2024 Sep 17;43(6):556–69. doi: 10.1002/zoo.21868 (PMC11624627; doi:10.1002/zoo.21868)
Supplement: Supplementary file 5 — Supplemental Figure 3 | Demographic of survey respondents. (A) Percentage of survey respondents that hold or have held venomous snakes at their institute. (B) Percentage of VS Holders and Non‐Holders that hold non‐venomous snakes at their organization. [file ZOO-43-556-s001.pdf]

Supplemental Figure 3.

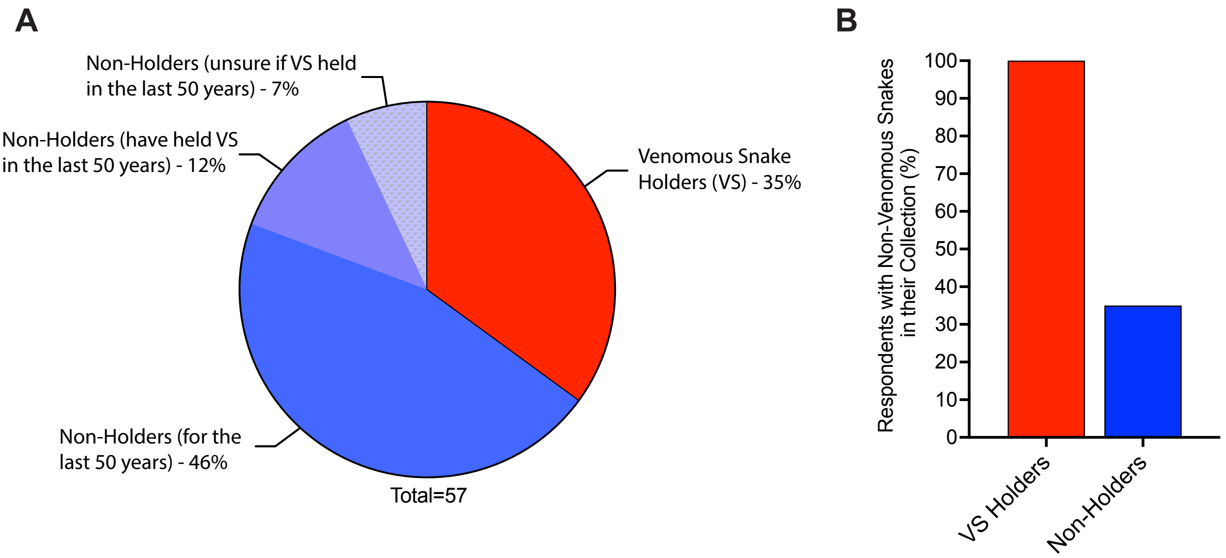

Supplemental Figure 3. Demographic of survey respondents. (A) Percentage of survey respondents that hold or have held venomous snakes at their institute. (B) Percentage of VS Holders and Non-Holders that hold non-venomous snakes at their organization.
